# When tasty becomes threatening: a neurobehavioral perspective on subjective food preferences in eating disorders

**DOI:** 10.3389/fpsyg.2026.1844251

**Published:** 2026-07-07

**Authors:** Annakarina Mundorf, Annabelle Siebert, Christina von der Assen, Christian Brünahl, Jutta Peterburs

**Affiliations:** 1 Institute for Systems Medicine and Department of Human Medicine, MSH Medical School Hamburg, Hamburg, Germany; 2 Department of Psychology, MSH Medical School Hamburg, Hamburg, Germany; 3 Department of Medicine, Psychosomatic Medicine and Psychotherapy, MSH Medical School Hamburg, Hamburg, Germany; 4 Clinic of Psychosomatic Medicine and Psychotherapy, Helios Clinics Schwerin, Schwerin, Germany

**Keywords:** anorexia nervosa, binge eating disorder, bulimia nervosa, food perception, reward processing, subjective preferences

## Abstract

Subjective food preferences significantly influence eating behavior, and in eating disorders (EDs), these preferences are often altered. Neuroimaging and neurobehavioral studies have revealed atypical brain responses to food cues in anorexia nervosa (AN), bulimia nervosa (BN), and binge eating disorder (BED), and these findings may point to disrupted subjective food valuation as a key neurobiological mechanism. This narrative review synthesizes recent findings suggesting that altered reward processing and valuation contribute to the persistence and complexity of disordered eating behaviors. The review is based on a comprehensive search of PubMed and additional citation searching, with study selection guided by conceptual relevance to subjective food valuation, reward processing, and decision-making in EDs. The review begins by outlining the conceptual foundations of subjective valuation and preference formation, followed by empirical evidence of altered reward processing and neural correlates of food valuation in AN, BN, and BED. Studies in clinical populations showing disrupted reward learning and maladaptive food choices are integrated to illustrate disorder-specific patterns. Across EDs, converging evidence indicates disorder-specific alterations in subjective food valuation and reward processing. While AN is characterized by reduced hedonic valuation, diminished reward responsiveness, and increased reliance on cognitive control mechanisms, BN and BED are more strongly associated with heightened hedonic and motivational salience of food cues, alongside dysregulated reward learning and variable engagement of cognitive control systems. The review concludes with a discussion of broader implications, future research directions, and potential clinical applications that emphasize integrating subjective preferences into ED research and treatment. Investigating EDs as disorders of disrupted subjective food valuation offers a unifying neurobehavioral perspective that expands our understanding of ED psychopathology and may lay the groundwork for targeted interventions addressing maladaptive reward processing.

## 1 Introduction

Eating disorders (EDs) are psychiatric conditions characterized by persistent disturbances in eating behavior that lead to clinically significant impairments in physical health and psychosocial functioning, as defined by the DSM-5 (American Psychiatric Association, 2013). They are often associated with disturbances in body image and maladaptive cognitive beliefs about food and eating. EDs affect millions of individuals worldwide. An estimated 13.6 million people are living with anorexia nervosa (AN) alone, and this disorder accounts for the majority of ED-related deaths (GBD 2019 Mental Disorders Collaborators, 2022). Despite lower prevalence compared to other psychiatric conditions, EDs are among the most chronic and deadly, especially for women (8.4% lifetime prevalence vs. 2.2% in men; Galmiche et al., 2019). The DSM-5 defines three main EDs relevant to the present review: AN, bulimia nervosa (BN), and binge eating disorder (BED). AN involves underweight and malnutrition and is further classified into two subtypes: the restricting type, marked by weight loss primarily through dieting, fasting, or excessive exercise, and the binge-eating/purging type, which involves episodes of binge eating or purging behavior; BN is characterized by binge eating and compensatory behaviors; BED features uncontrolled overeating without purging (American Psychiatric Association, 2013; O’Loghlen et al., 2022). Beyond diagnostic criteria, these disorders are also characterized by distinct clinical presentations and lived experiences (Wonderlich et al., 2021). Individuals with AN often exhibit intense fear of weight gain and high levels of food-related anxiety, whereas BN and BED are more strongly associated with loss-of-control eating and dysregulated patterns of intake (US Preventive Services Task Force et al., 2022). Importantly, although these disorders share disturbances in eating behavior, they differ substantially in their behavioral expression, physiological consequences, and associated patterns of cognitive and affective control (US Preventive Services Task Force et al., 2022). These diagnostic differences are also reflected in variability across appetite regulation, cognitive control, and reward-related responding (Wonderlich et al., 2021). Across AN, BN, and BED, food-related stimuli can elicit markedly different behavioral and affective responses, including altered reward responsiveness and heightened anxiety in AN, and more cue-driven or dysregulated eating patterns in BN and BED (Jiang et al., 2010; Ralph-Nearman et al., 2019; Wonderlich et al., 2021). Notably, while these differences suggest variability in underlying mechanisms, they do not map directly onto any single explanatory framework (Wonderlich et al., 2021). These findings suggest that uniform accounts of altered food-related valuation may be insufficient to capture ED-specific differences in motivational and hedonic processing fully. Within this context, subjective food valuation may provide a potentially useful transdiagnostic dimension that complements existing diagnostic frameworks by focusing on how food-related reward and motivation are computed and experienced across ED presentations.

Neuroimaging studies have reported divergent patterns of brain activation in individuals with AN, BN, and BED in response to food stimuli, particularly in regions implicated in reward processing, interoception, and cognitive control (Ralph-Nearman et al., 2019; Wonderlich et al., 2021; Leenaerts et al., 2022; Vrieze and Leenaerts, 2023). Several complementary theoretical frameworks have been proposed to account for these heterogeneous findings. These frameworks can be organized into complementary levels of explanation. First, neurobiological reward models propose that altered responsivity of mesolimbic and striatal circuits contributes to atypical reward experiences in EDs. For example, the *Neurobiological Reward Deficit Model* suggests reduced responsiveness to food reward in AN (Frank et al., 2012, 2021). Second, reinforcement learning and reward prediction error frameworks emphasize how discrepancies between expected and received outcomes shape maladaptive learning, anticipation, and decision-making processes (Schultz, 2016; Schaefer and Steinglass, 2021). Third, behavioral reinforcement accounts distinguish between impulsive, cue-driven overeating and more compulsive, habit-based behaviors, highlighting how maladaptive eating may become increasingly automated through reinforcement learning processes (Burger, 2023). Across these frameworks, a central construct is the distinction between anticipatory motivation (“wanting”) and consummatory pleasure (“liking”; Berridge and Robinson, 2016; Morales and Berridge, 2020). While this framework has been applied to EDs, empirical findings are heterogeneous, particularly in AN, where studies report both reduced and relatively preserved hedonic responses (Frank et al., 2012, 2021; Ralph-Nearman et al., 2019).

Building on this perspective, the present review offers a neurobehavioral lens on EDs, with a focus on subjective food valuation and the underlying reward mechanisms. Existing models and empirical studies have provided important insights into altered reward processing in EDs (Ralph-Nearman et al., 2019; Frank et al., 2021; Wonderlich et al., 2021). Research has clarified neural correlates of reward anticipation and consumption across AN, BN, and BED (Leenaerts et al., 2022; Vrieze and Leenaerts, 2023), identified transdiagnostic mechanisms such as reward prediction error signaling (Schaefer and Steinglass, 2021), and illuminated how motivational and hedonic processes can become dissociated (Berridge and Robinson, 2016; Morales and Berridge, 2020). However, these findings remain fragmented across paradigms, diagnostic categories, and levels of analysis, and key questions remain regarding how subjective food valuation is computed and integrated across behavioral, neural, and computational domains (Bodell and Racine, 2023; Burger, 2023). Rather than proposing an entirely new model, we aim to build on existing theoretical accounts by exploring how altered cognitive-emotional valuation processes may shape food-related behavior and treatment responses in EDs. To identify relevant studies, we conducted a comprehensive PubMed search complemented by citation searching of relevant articles and recent publications. Literature selection was guided by conceptual relevance to subjective food valuation, reward processing, and food-related decision-making in AN, BN and BED. Given this conceptual and methodological heterogeneity, findings are distributed across different levels of analysis and are often examined within specific diagnostic categories. In this context, a purely systematic synthesis may not fully capture the cross-level relationships between neural, behavioral, and computational accounts of reward processing. A narrative and integrative approach as applied for the present review is therefore particularly well suited to synthesize these diverse literatures and to develop a coherent framework focused on subjective food valuation in EDs.

To facilitate interpretation of the subsequent literature, the following section (2 Conceptual foundations of subjective food preferences and reward processing) first summarizes the theoretical and methodological foundations of subjective valuation and preference formation. We then examine empirical evidence for altered reward processing in EDs across behavioral, neural, and electrophysiological domains (3 Evidence from patient studies on reward processing and subjective preferences). Special attention is given to studies with clinical populations, including findings on disrupted reward learning and maladaptive food choices. Finally, we discuss the broader implications of these mechanisms for clinical practice and highlight conceptual and methodological avenues that may inspire future research in the field (4 Discussion).

## 2 Conceptual foundations of subjective food preferences and reward processing

Before reviewing alterations in reward processing in EDs, it is important to clarify several concepts that are central to the interpretation of food-related valuation and decision-making.

### 2.1 Concepts and relevance

To understand the complex neural mechanisms underlying EDs in general, it is essential to define three important concepts, *subjective valuation*, *hedonic value*, and *subjective preferences*, all of which play a crucial role in how individuals process and respond to food stimuli.

- *Subjective valuation* refers to the process by which individuals assign personal worth or importance to an object or experience based on emotional and psychological factors, rather than objective measures.
- *Hedonic value* specifically reflects the pleasure (or displeasure) derived from consuming food, influenced by both physiological and psychological factors. Hedonic responses may become altered, leading to food stimuli being perceived as aversive or anxiety-inducing rather than rewarding.
- *Subjective preferences* refer to an individual’s personal choices or inclinations, shaped by their unique experiences, emotions, and mental states. Subjective preferences in the realm of food can be considered trait-like in that they are relatively stable intra-individually but may differ between individuals (Rozin, 1990; Skinner et al., 2002; Nicklaus et al., 2004). For instance, a person may prefer white chocolate over dark chocolate or peanuts, while the reverse is true for another individual, and these preferences are stable over time.

Importantly, *subjective valuation* and *subjective preference* do not necessarily covary. In the present review, we focus primarily on subjective preferences, as they have been linked to neural mechanisms of observable food choices and decision-making (Gao et al., 2026). While hedonic value and subjective valuation provide context, preferences determine behavior (Rangel et al., 2008; Gao et al., 2026) and are therefore central to understanding EDs. While valuation reflects an internal assessment of worth or value, preference reflects a choice or inclination that may be influenced by, but is not synonymous with, valuation (Rangel et al., 2008; Gao et al., 2026). They are measured differently: subjective valuation is often assessed via self-report ratings of value or worth (e.g., visual analog scales, Likert-type items; e.g., Gibbons et al., 2019; Scheufele et al., 2025), whereas subjective preference is typically captured by choice-based paradigms such as paired comparisons, forced-choice tasks, or ranking procedures (e.g., (Finlayson et al., 2008; Cowdrey et al., 2013; Steinglass et al., 2015). These approaches differ in their elicitation format (rating vs. choice) and may therefore capture partially distinct aspects of decision-making. This distinction is consistent with work on implicit and explicit components of food reward, which suggests that rating-based and choice-based measures can be dissociated under certain conditions (Finlayson et al., 2008; Rangel et al., 2008; Levy and Glimcher, 2012). From a neuroeconomic perspective, these differences have been interpreted as reflecting partially separable components of value representation, rather than a single unified construct (Levy and Glimcher, 2012).

Importantly, differences between measurement approaches may be particularly relevant in clinical populations, where subjective ratings and behavioral choices do not always show strong convergence. This variability is consistent with evidence showing that emotional state and ED symptom severity can modulate reward-based food learning and bias formation (Rouhani et al., 2025), suggesting that preference-related behavior is not fixed but context-dependent.

Subjective preferences generally covary with behavioral preferences, such as observable choices made in experimental or real-world settings, though this relationship may not always be perfect. Behavioral preferences can be influenced by situational factors, availability, or social context, which may cause divergence from reported subjective preferences (Shepherd, 1999; Fernqvist et al., 2024; Jiao, 2024). This discrepancy has prompted the use of implicit measures, such as reaction-time-based tasks or eye-tracking, to assess motivational salience and implicit preferences, which may be more sensitive to unconscious or conflicted attitudes toward food in clinical groups. Explicit subjective preferences refer to consciously accessible and verbally reportable evaluations of food stimuli, whereas implicit subjective preferences reflect automatic motivational valuation processes that may guide behavior independently of reflective endorsement (Morales and Berridge, 2020). Neuroeconomic models propose that subjective value is represented within a common neural currency in the ventromedial prefrontal cortex (vmPFC)/ orbitofrontal cortex (OFC; Levy and Glimcher, 2012). At the same time, separable components of reward processing, such as “liking” and “wanting,” may differentially contribute to experienced versus motivational aspects of preference (Morales and Berridge, 2020).

Methodologically, the investigation of these constructs has advanced significantly through the use of neuroimaging (Gao et al., 2026). Event-related fMRI designs are ideal for assessing brain activation in response to discrete food stimuli and allow for dissociating anticipatory (cue-related) from consummatory (taste-related) phases of reward processing (e.g., Small et al., 2001; Tomova et al., 2020). Meanwhile, EEG offers superior temporal resolution and has been used to track rapid neuronal responses to food cues, e.g., in event-related potentials associated with attention and affective evaluation (e.g., Banica et al., 2023; Huvermann et al., 2021; Peterburs et al., 2019; Tashiro et al., 2019). A combination of EEG and fMRI offers complementary insights, offering both high temporal resolution and precise localization of neural responses. This multimodal approach is particularly promising for elucidating altered valuation and preference mechanisms in ED populations, where subtle temporal and spatial changes might coexist.

Subjective preferences are influenced by a complex interplay of genetic predispositions (Koenders et al., 2024), early life experiences, and repeated exposure (Wadhera et al., 2015; Scaglioni et al., 2018), which shape neural circuits related to reward and memory. Because many of these influences tend to be consistent over time, subjective preferences often remain relatively stable within an individual, forming a kind of trait-like characteristic (Rozin, 1990; Skinner et al., 2002; Nicklaus et al., 2004). Stability is also reinforced by learned associations. For example, persistent positive experiences with a certain food confirm its hedonic value, maintaining it as a preferred choice in future encounters. Additional contributing mechanisms include flavor-nutrient conditioning (de Araujo et al., 2013) and interoceptive reinforcement learning based on post-ingestive signals (Weber et al., 2025).

### 2.2 Neural mechanisms in healthy individuals

Neuroimaging research indicates that subjective food preferences are dynamically encoded in reward-related brain regions, including the ventral striatum, OFC, insula, and midbrain, and are modulated by physiological states such as hunger or satiety (Small et al., 2001; Tomova et al., 2020). These neural representations are thought to support the valuation processes that guide food-related decisions and behavioral choices. Through repeated experiences, reward-related responses contribute to the formation of associative memory traces linking specific foods with expected outcomes such as pleasure or satiety. Electrophysiological markers, including the Feedback-Related Negativity (FRN) and Reward Positivity (RewP), similarly reflect neural responses to positive outcomes and reward prediction, tracking the brain’s sensitivity to preferred foods (Sambrook and Goslin, 2015; Von der Assen et al., 2026). Together, these studies highlight that subjective valuation of food influences both anticipatory and consummatory neural processing, and that motivational states interact with preferences to shape behavior. Collectively, these findings support the view that subjective food valuation emerges from the interaction of reward learning, memory, and state-dependent modulation rather than reflecting a static preference signal. Briefly, studies by Peterburs et al. (2019) and Huvermann et al. (2021) demonstrate that neural responses to food rewards are stronger for preferred items and change with prior consumption, illustrating that subjective preference and contextual factors dynamically modulate reward processing. These findings in healthy populations provide a framework for understanding EDs, where reward processing and subjective food valuation may be dysregulated, setting the stage for examining how these mechanisms manifest in clinical populations.

## 3 Evidence from patient studies on reward processing and subjective preferences

Building on the conceptual framework outlined above, empirical studies in clinical populations have examined how alterations in reward processing and subjective food valuation manifest across EDs.

In individuals with EDs, the neural and behavioral mechanisms supporting subjective food preferences are often disrupted. Previously pleasurable foods may acquire aversive or anxiety-inducing properties due to heightened fear responses, cognitive biases, and maladaptive beliefs about food and body image (Small et al., 2001; Joos et al., 2011; Cowdrey et al., 2013; Stover et al., 2023). These alterations may interfere with normal reward learning and decision-making, leading to persistent restrictive or binge-related behaviors. Feedback learning deficits, impaired integration of anticipatory and consummatory signals, and habitual stimulus–response processes may further reinforce these maladaptive preferences (Foerde and Steinglass, 2017; Mueller et al., 2018; Schaefer and Steinglass, 2021). Importantly, evidence across studies suggests that subjective food preferences in EDs cannot be fully explained by altered hedonic responses alone, but likely reflect a broader disruption in reward learning, value updating, and cognitive control processes.

To ensure conceptual coherence, studies reviewed in the present work were selected and organized based on their relevance to core constructs of reward valuation, decision-making, and food-related behavior rather than exhaustive coverage of all available literature. Because ED diagnoses differ substantially in their behavioral phenotype, most notably in the contrast between restrictive eating patterns in anorexia nervosa and binge-eating behaviors in bulimia nervosa and binge-eating disorder, findings are summarized separately by diagnosis. This approach allows disorder-specific patterns in neural and behavioral responses to food-related rewards to be identified.

### 3.1 Anorexia nervosa

**Figure 1:**
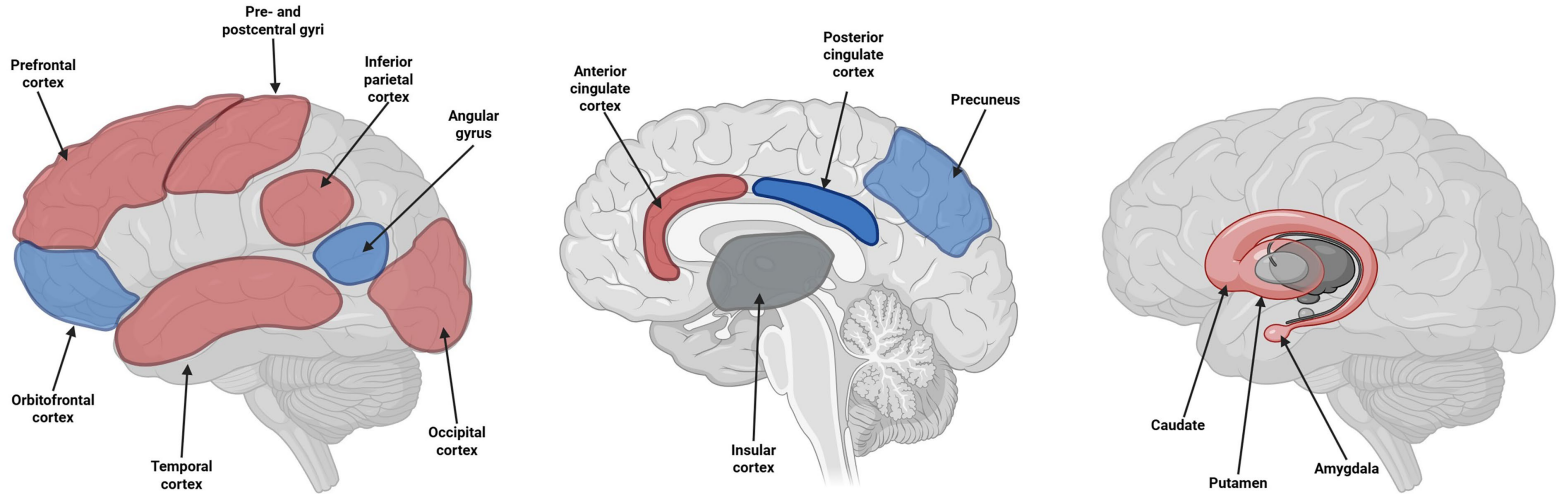
Neural correlates of food-related processing in anorexia nervosa. This schematic illustration summarizes neuroimaging findings in individuals with anorexia nervosa across functional magnetic resonance imaging studies. Brain regions are grouped by anatomical location and highlighted according to the predominant direction of neural activity differences observed relative to comparison groups, including healthy control participants. Blue shading indicates decreased activity, while red shading indicates increased activity in individuals with anorexia nervosa. Regions with heterogenous findings are marked in gray. Color saturation reflects the frequency with which alterations in a given region were reported across studies, with darker shades indicating more frequently reported findings and lighter shades indicating regions supported by fewer studies. Regions depicted include the orbitofrontal cortex (medial and lateral portions), caudate nucleus, putamen, anterior cingulate cortex, insular cortex, prefrontal cortex, supplementary motor area, precentral and postcentral gyri, occipital cortex, posterior cingulate cortex, precuneus, angular gyrus, and amygdala. Created in BioRender. Mundorf, A. (2026) https://BioRender.com/lho2jq4.

In AN, studies have documented reduced pleasure and altered neural responses to food cues, highlighting atypical subjective valuations. Building on these findings, more recent research has explored food choice behavior, reward prediction errors, and memory-related mechanisms, showing that altered caudate, orbitofrontal, and striatal responses are linked to restrictive food selection and persistent avoidance (for a summary of key findings, see Table 1 and Figure 1). For example, Santel et al. (2006) found that individuals with AN rated food stimuli as less pleasant than healthy controls. Furthermore, participants with AN showed reduced activation in the left inferior parietal cortex when satiated compared to controls. When hungry, they exhibited weaker activation in the right visual occipital cortex than controls. Food stimuli during satiety compared to hunger triggered stronger activation in the right occipital cortex in affected subjects, and in the left lateral OFC, middle right anterior cingulate, and left middle temporal gyrus in controls (Santel et al., 2006). This pattern suggests that subjective food preferences in AN are mirrored in reduced engagement of neural circuits typically involved in attentional and sensory processing of appetitive stimuli.

**Table 1:** Summary of findings from patient studies on reward processing and subjective preferences in anorexia nervosa.

	Author, year	Paradigm	Groups compared in study	Sample (Gender, N, Age in years)	Illness characteristics (Duration, severity, recovery status, treatment/medication	Neuroimaging results
Reward processing and subjective preferences	Santel et al. (2006)	High calorie food images and images of objects satiated	AN-ill, HC	23 females AN-ill: 13, HC: 10, mean age AN-ill: 16.1 ± 2, mean age HC: 16.8 ± 2.6	Time since first onset median: 8 months [3–62 months], 10 AN first episode < 1 year, inpatients SSRI (4 AN)	satiated AN: ↓ in left inferior parietal cortex relative to HC; hungry AN: ↓ right visual occipital cortex relative to HC; food stimuli during satiety compared with hunger were associated with ↑ right occipital in AN
	Gizewski et al. (2010)	High vs. low calorie food images while being hungry	AN-ill, HC	24 females AN-ill: 12, HC: 12; mean age AN-ill: 27 [18–52], mean age HC: 21 [21–35]	Duration of illness: 84.5 8 ± 42.8 months, inpatients free of medication for at least 3 months	hungry: HC: ↑ ACC and insula, AN: ↑ prefrontal + postcentral cortices + insula + ↓ in right visual occipital cortex; satiated: ↑ left inferior parietal cortex in AN to HC
	Joos et al. (2011)	Viewing pictures of food and non-food items	AN-R, HC	22 females AN-R: 11, HC: 11, mean age AN-R: 25 ± 5, mean age HC: 26 ± 5.2	Duration of illness: 5.0 ± 3.6 years 1 AN on sertraline 75 mg/day	↑ in right amygdala + ↓ posterior midcingulate cortex in AN-R relative to HC; AN: ↑ frontolimbic regions + precuneus
	Brooks et al. (2011) #	Imagining eating food shown in images or using non-food items	AN-R, AN-BP, BN, HC	50 females AN-R: 11, AN-BP: 7, BN: 8, HC: 24 mean age BN: 25 ± 7.1, mean age AN: 26 ± 6.8 mean age HC: 26 ± 9.5	AN: inpatient unit, average duration of illness AN-R: 9 years, AN-BP: 9 years BN: outpatient center, average duration of illness: 12 years antidepressant medication included	compared to HC: AN: ↓ in parietal lobe + dorsal posterior cingulate cortex, ↑ in caudate, superior temporal gyrus, right insula + supplementary motor area
	Boehm et al. (2021)	Viewing streams of food stimuli, social stimuli, and neutral stimuli	AN-ill, AN-rec, HC	126 females AN-ill: 35; recAN: 33; HC: 58 mean age: 15,0–29.0	AN-ill: admitted to ED programs of psychiatry and psychosomatic medicine department AN-rec: had to maintain a BMI > 18.5 kg/m^2^ for at least 6 months prior to the study HC: no former ED diagnosis or other clinical/medical conditions	AN-ill: ↑ classification accuracy for food stimuli in the right posterior fusiform gyrus compared to HC
	Olsavsky et al. (2019)2020 #	Associative taste reward learning paradigm	AN-ill, AN-rec, BN, HC	111 females AN-ill: 28, AN-rec: 20, BN: 20, HC: 43 mean age AN-ill: 22.9 ± 5.0, mean age AN-rec: 30.0 ± 8.0, mean age BN: 25.3 ± 4.6, mean age HC: 26.4 ± 5.4	AN + BN: admitted to specialized treatment program, studied during first 1–2 weeks of treatment antidepressants (14 AN-ill, 4 AN-rec, 13 BN), antipsychotics (3 AN-ill, 3 BN)	AN-ill: ↑ expected value signal in left ACC compared to AN-rec, HC
Reward processing and subjective preferences	Jiang et al. (2019) #	Liking and wanting tasks using food odors	AN-R, BN, HC	39 females ANR: 14; BN: 13; HC: 12 mean age AN-R: 24.9 ± 4.7 mean age BN: 22.5 ± 2.9 mean age HC: 24.1 ± 3.1	AN-R: hospitalized BN: purging and binge-eating behaviors but ambulatory.	Wanting (motivational valuation) AN-R vs. HC ↓ VTA, ventral pallidum, anterior insula, precuneus, Liking (hedonic evaluation) AN-R vs. HC ↓ Ventral pallidum and insula Hunger modulation HC: ↑ activity in reward regions AN-R: ↓ blunted hunger modulation
Disrupted reward learning and food choice	Foerde et al. (2021)	Food choice task	AN-ill, HC	53 females AN-ill: 24 (AN-R: 9, AN-BP: 15), HC: 29 mean age AN: 26.9 ± 6.5, mean age HC: 25.8 ± 5.2	Hospitalized, weight restoration, no medication	AN ↓ caudate activity associated with ↑ selection of high-fat foods compared to HC
	Xue et al. (2022)	Food choice deliberation task	AN-ill, HC	41 females AN-ill: 20 (10 AN-R + 10 AN-BP), HC: 21 mean age AN-ill: 26.4 ± 6.5, mean age HC: 22.7 ± 3.1	Inpatients, no medication	AN: ↓ lateral + medial OFC compared to HC
	Frank et al. (2012) #	Reward-conditioning task	AN-R, Obese, HC	63 females AN-R: 21, obese: 19, HC: 23 mean age AN-R: 22.5 ± 5.8, mean age obese: 27.1 ± 6.7, mean age HC: 24.8 ± 5.6	Illness duration AN: 6.48 ± 5.29 years Obese: 11.25 ± 5.75 years SSRI + atypical antipsychotics (2 AN), SSRI (6 AN), atypical antipsychotics (2 AN)	AN: ↑ in left OFC vs. both HC and obese; ↑ in right cingulate and medial frontal cortex vs. obese; ↑ in putamen + SMA vs. obese; ↓ during negative prediction error vs. HC + obese; ↓ in right SMA during sucrose expectation vs. HC and obese ↓ negative activation during negative prediction error vs. AN
	Gorrell et al. (2024)	Taste conditioning and expectation-reward task	AN-ill, AN-rec	35 females mean age: 23.0 ± 7.0	All participants received ED treatment in specialized partial hospital or residential program Medications: antidepressants (*n* = 14), antipsychotics (*n* = 6), mood stabilizers (*n* = 2), and stimulants (*n* = 3).	↑ activity in OFC, ventral striatum and nucleus accumbens during expectation of caloric reward predicted lower long-term BMI (worse outcome) ↑ activity in caudate by involvement in action-outcome learning and habitual processing ↑ anticipatory OFC / striatal activation → lower BMI at follow-up
Disrupted reward learning and food choice	Weider et al. (2024)	Goal value decision task (food avoidance/aversion)	AN, HC	49 females AN: 19, HC: 30 mean age AN: 18.3 ± 5.9 mean age HC: 21.4 ± 6.0	AN: hospitalized, weight restoration, no other ED, right-handed without a history of head trauma, neurological disease, major medical illness, bipolar disorder, psychosis, or current (past 3 months) substance use disorder HC: no lifetime psychiatric or other illness, no ED disorder history, no first-degree relative with an ED, normal lifetime BMI	AN (compared to HC): ↑ Caudate nucleus in aversive food avoidance ↑ Nucleus accumbens in decisions to avoid food ↑ vACC ↑, OFC ↑ during target value assessment ↑ Higher bids for avoidance → Caudate nucleus ↑ ↑ lower BMI → nucleus accumbens ↑ (stronger aversive value) ↓ nucleus accumbens, caudate, vACC in emotional dysregulation/intolerance of uncertainty
	Terhoeven et al. (2023)	Specific food/body-related memory vs. example generation	AN-ill, HC	59 females AN-ill: 29; HC: 30 mean age AN-ill: 23.2 ± 4.9, mean age HC: 23.7 ± 3.7	Duration of illness: 6.1 ± 5.8 years Antidepressants (1 AN) + neuroleptics (4 AN)	↓ recall of specific food/body-related memories; ↓ in precuneus + angular gyrus compared to HC; ↑ in precentral gyrus for neutral memory recall vs. HC; ↑ in precentral and middle temporal gyrus during general food vs. neutral recall vs. HC; ↓ connectivity in the posterior cingulate cortex + middle frontal gyrus than HC

Included studies compare findings from functional magnetic resonance imaging in patients with anorexia nervosa with healthy controls. The table includes details on sample composition and illness characteristics. In the “Neuroimaging results” column, arrows are used to summarize group differences in brain activation or behavioral responses: an upward arrow (↑) indicates increased activation or preference in the patient group compared to controls, a downward arrow (↓) indicates decreased activation or preference, and a bidirectional arrow (↔) denotes no significant difference between groups. # A subgroup of patients with bulimia nervosa or binge-eating disorder was included; the respective findings are summarized in Table 2. ACC, anterior cingulate cortex; AN-ill, anorexia nervosa, currently ill; AN-R, anorexia nervosa restricting subtype; AN-BP, anorexia nervosa binge purging; AN-rec, anorexia nervosa, recovered; dlPFC, dorsolateral prefrontal cortex; dmPFC, dorsomedial prefrontal cortex; ED, eating disorder; HC, healthy control; PCC, posterior cingulate cortex; PFC, prefrontal cortex; OFC, orbitofrontal cortex; SMA, supplemental motor area; VTA, ventral tegmental area.

In a similar vein, Gizewski et al. (2010) found that during hunger, healthy participants showed significant activation in the anterior cingulate cortex (ACC), insula, and posterior cingulate cortex (PCC) in response to food stimuli. In contrast, subjects with AN exhibited increased activation in the PFC, postcentral gyrus, and bilateral anterior insula. When satiated, subjects with AN showed decreased activation in the left inferior parietal cortex compared to controls, and weaker activation in the right visual occipital cortex during hunger. Food stimuli during satiety compared to hunger were associated with stronger activation in the right occipital cortex in participants with AN, and in the left lateral OFC, middle right anterior cingulate, and left middle temporal gyrus in healthy controls (Gizewski et al., 2010). These findings suggest that neural responses to high-calorie food images are modulated not only by hunger or satiety but also by subjective food valuation, with cognitive control regions in patients with AN potentially overriding interoceptive hunger cues. Using food valence as a covariate confirmed insula activation in both groups and revealed additional activation in the orbitofrontal, cingulate, and medial temporal cortices. This suggests that altered subjective valuation in AN may influence how food-related stimuli are processed, with cognitive control regions playing a critical role in modulating these neural responses.

Collectively, these findings converge on the notion that altered food valuation in AN is not limited to reduced reward responsiveness but involves a broader reorganization of attentional, sensory, and control-related processing during food evaluation. This aligns with findings by Joos et al. (2011), who observed elevated activity in the right amygdala of participants with restrictive subtype AN when viewing food images compared to controls. Based on the functional topography of the amygdala (Ocklenburg et al., 2022), this may point to an emotionally charged, potentially ambivalent or threat-based appraisal of food-related cues in participants with AN of the restrictive subtype. Using a disorder-specific paradigm, Joos et al. (2011) also found increased activity in frontolimbic regions and the precuneus in subjects with AN and decreased activation in the posterior midcingulate cortex in AN compared to controls. The combination of increased amygdala activity and decreased cingulate activity may reflect a negative feedback loop in emotional processing, with disgust ratings negatively correlating with amygdala activity, highlighting the complex role of this brain region in EDs.

Brooks et al. (2011) reported that participants with AN exhibited increased activation in the cerebellum, right dorsolateral PFC (dlPFC), and right precuneus when viewing food images, suggesting heightened visual processing and engagement of executive control networks. Compared with healthy controls, AN patients also showed deactivation in the parietal lobe and dorsal PCC, but greater activation in the caudate, superior temporal gyrus, right insula, and supplementary motor area (Brooks et al., 2011). These patterns indicate that AN involves strong top-down cognitive control over food-related stimuli, potentially reflecting effortful regulation of motivation and attention toward food cues.

Boehm et al. (2021) extended these findings by comparing acutely ill and weight-recovered AN participants. Acutely ill patients showed altered neural representation of food stimuli in secondary visual areas, with greater classification accuracy of food images compared to healthy controls, whereas weight-recovered individuals did not differ from controls. Importantly, the degree of altered neural representation in acutely ill AN was associated with better treatment outcome, suggesting that increased attentional engagement with food stimuli may paradoxically facilitate recovery (Boehm et al., 2021). These findings emphasize the role of subjective food processing in AN and suggest that visual attention and neural representation of food may be a key feature of illness severity and recovery potential.

Olsavsky et al. (2019) extended these findings into the domain of reward learning, showing that acutely ill AN participants exhibited stronger expected value signals in the ACC during an associative taste reward task compared with recovered AN, BN, and healthy controls. This suggests that the subjective valuation of food rewards is altered in acute AN, possibly contributing to restrictive eating behavior. Notably, intolerance of uncertainty negatively correlated with expected value signals in acute AN, highlighting a potential mechanism by which heightened sensitivity to prediction errors or uncertainty reinforces rigid food restriction (Olsavsky et al., 2019). Together, these studies suggest that AN is characterized by a combination of heightened cognitive control and altered reward valuation in response to food stimuli.

Generally, these results suggest that in AN, reward-related alterations are closely intertwined with enhanced cognitive control and atypical integration of learning and memory processes, rather than reflecting a uniform reduction in reward sensitivity. Consistent with models proposing diminished reward sensitivity in AN, Jiang et al. (2019) reported reduced motivational responses to food cues in individuals with the restrictive subtype. Behaviorally, patients with AN indicated a lower desire to eat odor-cued foods, reflecting attenuated subjective “wanting.” On the neural level, AN patients exhibited reduced activation of the ventral tegmental area (VTA) during the wanting task when exposed to food odors. Given the VTA’s central role in dopaminergic signaling and incentive salience attribution, this finding suggests blunted assignment of motivational value to food-related stimuli. Direct group comparisons indicated that this attenuation differentiated AN from BN, highlighting subtype-specific alterations within mesolimbic circuitry. Additionally, AN patients showed reduced activation in the anterior ventral pallidum and insula in response to high energy-dense food odors during hunger, pointing to altered integration of motivational and interoceptive signals. Lower precuneus activation further distinguished AN and may relate to disrupted self-referential processing or body image distortion during food evaluation (Jiang et al., 2019).

Building on these findings, more recent research has employed food choice tasks, reward prediction error paradigms, and memory-based assessments to investigate the neural and behavioral mechanisms underlying restrictive eating patterns. These studies provide a deeper understanding of how altered reward processing reinforces maladaptive food avoidance (see Table 1). Foerde et al. (2021) investigated subjective food preferences in individuals with AN using a food choice task during fMRI. Compared to controls, individuals with AN showed greater choice-related activation in the right caudate, particularly when evaluating high-fat foods, suggesting altered decision-making processes related to food. Although overall food choices and brain activity did not significantly change after weight restoration, decreases in caudate activation were associated with increased selection of high-fat foods, indicating a potential link between neural mechanisms and changes in food preference over time (Foerde et al., 2021). Similarly, Xue et al. (2022) investigated how food-related attributes are neurally represented during a food choice deliberation task in participants with AN and healthy controls using fMRI. Whole-brain searchlight analysis revealed that tastiness information was decodable from more brain regions in controls than in AN, including the lateral and medial OFC, suggesting reduced neural encoding of taste in AN. While both groups showed OFC involvement, health-related neural patterns in the OFC were more strongly associated with choice preferences in AN, indicating that maladaptive food decision-making in AN may involve an overemphasis on health considerations during deliberation (Xue et al., 2022). In a complementary study, Frank et al. (2012) focused on reward learning and prediction error responses in AN. Using a classical conditioning paradigm with sucrose solutions, they observed heightened activation in the anteroventral striatum and left OFC during positive prediction error (i.e., when outcomes were better than expected, such as receiving sucrose unexpectedly), as well as more negative activation across several brain regions during negative prediction error (i.e., when outcomes were worse than expected, such as an expected sucrose reward being omitted) in participants with AN, compared to both obese and healthy controls. Additionally, AN participants showed reduced activation in the right supplemental motor area during sucrose expectation compared to both groups. These findings suggest that individuals with AN exhibit altered responses during reward prediction and receipt, reflecting potentially disrupted reinforcement learning mechanisms. This contrasts with prior studies focusing on subjective preference or devaluation and advances the current understanding of maladaptive food avoidance in AN by highlighting the role of altered reward prediction error signaling and associated hypoactivation of related brain regions in food-related decision-making (Frank et al., 2012).

Across paradigms probing choice behavior, prediction error learning, and decision-making, a consistent pattern emerges in which food-related valuation in AN is increasingly shaped by cognitive and inferential processes rather than primary hedonic drive. More recent studies extend these findings to outcome prediction and food avoidance. Gorrell et al. (2024) used a taste conditioning paradigm to test whether neural responses to caloric stimulus expectation predicted long-term body mass index in women with AN. Greater activation in orbitofrontal and striatal regions during expectation, but not during reward receipt, was associated with lower body mass index and less weight gain at follow-up (Gorrell et al., 2024). This suggests that heightened anticipatory responses to food cues may reinforce food avoidance and long-term underweight status, highlighting the role of learned associations in maladaptive eating.

Similarly, Weider et al. (2024) employed a food avoidance bidding task to examine aversive goal value processing in AN. Individuals with AN showed greater activation in the caudate nucleus, nucleus accumbens, anterior cingulate, and medial OFC when placing higher bids to avoid food, reflecting heightened neural representation of food as an aversive stimulus (Weider et al., 2024). Brain responses were inversely related to emotional dysregulation and intolerance of uncertainty, indicating that these neural patterns may mediate the interaction between negative affect and restrictive behavior. Together, these findings provide a mechanistic link between altered reward and aversion processing and the maintenance of food avoidance in AN.

Shifting focus from current food preferences and decision-making, Terhoeven et al. (2023) examined how food-related autobiographical memories are cognitively and emotionally represented in individuals with AN. Using a modified memory task during fMRI, participants were asked to recall personal experiences related to food and body cues. While healthy participants recalled more specific food-related memories than participants with AN, those with AN showed reduced activation in areas such as the angular gyrus and precuneus, typically involved in memory retrieval, particularly during the recall of specific food-related memories. Additionally, functional connectivity was reduced in the PCC and middle frontal gyrus in subjects with AN compared to controls. Conversely, subjects with AN exhibited increased activation in the precentral and middle temporal gyri, especially during general food-related memory recall, suggesting a more effortful or differently organized memory retrieval process. This study highlights how disruptions in accessing emotionally relevant food-related memories may contribute to the avoidance and altered valuation of food in AN, offering a cognitive perspective on the mechanisms behind disrupted food-related preferences (Terhoeven et al., 2023).

These findings further indicate that anticipatory processes, aversive valuation, and autobiographical memory representations jointly contribute to the maintenance of restrictive eating, highlighting the multidimensional nature of altered food valuation in AN. Overall, individuals with AN exhibit altered neural processing of food stimuli across visual, attentional, emotional, and reward domains. Subjective valuation of food is diminished, reflected in reduced “wanting” ratings and attenuated activation in reward regions such as the ventral striatum, insula, and VTA. Enhanced engagement of cognitive control regions (dlPFC, ACC) and altered visual processing may reflect compensatory mechanisms. Studies using food choice, reward prediction, and autobiographical memory paradigms indicate disruptions in reinforcement learning, decision-making, and memory-related food representations, which may reinforce restrictive behaviors. Collectively, these findings highlight a complex interplay of diminished hedonic value, disrupted reward learning, altered incentive salience, and heightened cognitive control, supporting the view of AN as a disorder of impaired subjective food valuation. Further research is needed to clarify interactions across neural systems, differences across subtypes, and links to recovery trajectories.

### 3.2 Bulimia nervosa and binge-eating disorder

**Figure 2:**
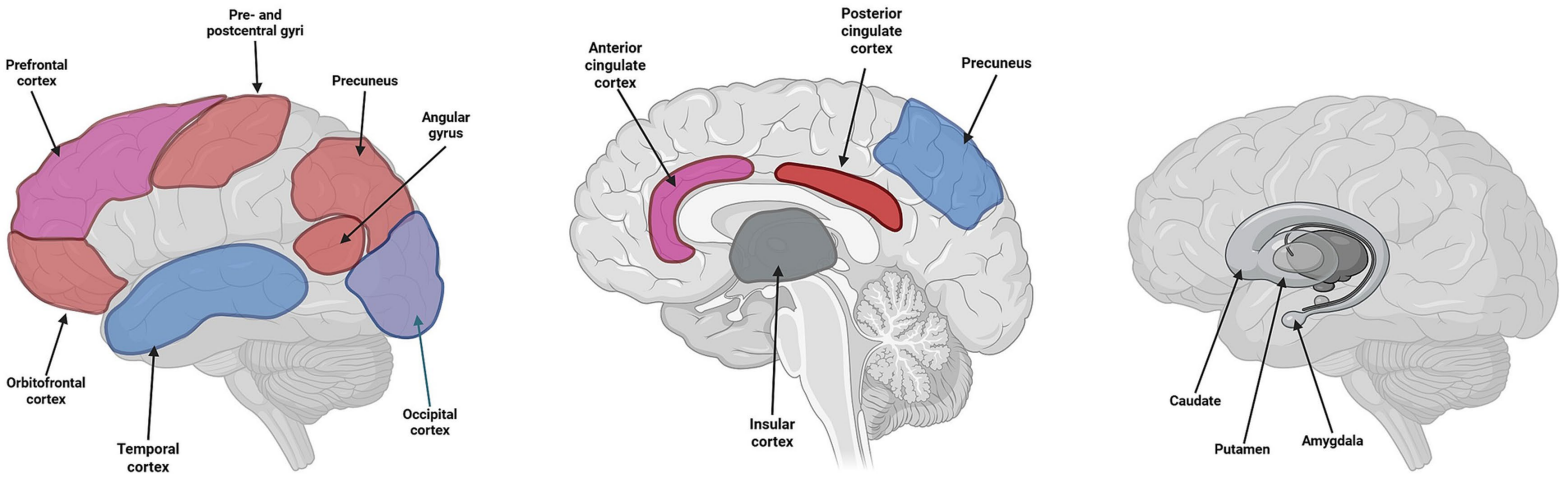
Neural correlates of food-related processing in bulimia nervosa and binge-eating disorder. This schematic illustration summarizes neuroimaging findings in individuals with bulimia nervosa and binge-eating disorder across functional neuroimaging studies. Brain regions are highlighted according to the predominant direction of neural activity differences observed relative to comparison groups, including healthy control participants and other clinical groups, depending on the study design. Red indicates increased activity relative to comparison groups, whereas blue indicates decreased activity. Dark red and dark blue denote regions reported in both bulimia nervosa and binge-eating disorder, while pink and purple denote regions reported in only one of the two disorders. Color saturation reflects the frequency of evidence across studies. Regions with heterogenous findings are marked in gray. Regions shown include the orbitofrontal cortex (particularly medial portions), anterior cingulate cortex, insular cortex, occipital cortex, posterior cingulate cortex, and angular gyrus. Created in BioRender. Mundorf, A. (2026) https://BioRender.com/9ha2rr6.

In contrast to AN, where restrictive eating and altered reward prediction dominate, individuals with BN and BED exhibit heightened hedonic responses and motivational salience to food cues, which shape binge-eating behavior (Small et al., 2001; Joos et al., 2011; Simon et al., 2016). Research in BN and BED has therefore focused on neural and behavioral responses to food cues, examining how hedonic valuation and motivational processes contribute to eating behaviors such as binge episodes (for an overview of relevant studies, see Table 2 and Figure 2).

**Table 2:** Summary of findings from patient studies on reward processing and subjective preferences in bulimia nervosa and binge-eating disorder.

	Author, year	Paradigm	Groups compared in study	Sample (Gender, N, Age in years)	Illness characteristics (Duration, severity, recovery status, treatment/medication	Neuroimaging results
Reward processing and subjective preferences	Schienle et al. (2009)	Condition: visual exposure to high-caloric food	BN, BED, obese HC, normal weight HC	67 females, BN: 14, BED: 17, overweight HC: 17, normal-weight HC: 19 mean age BN: 23.1 ± 3.8, mean age BED: 26.4 ± 6.4, mean age overweight HC: 25 ± 4.7, mean age HC: 22.3 ± 2.6	Duration of illness: BN: 7.3 ± 3.6 years BED: 6.8 ± 4.0 years No medication	BN: ↑ ACC + insula compared to BED + both HC groups; BN: ↑ arousal; BED: ↑ medial OFC + ↑ reward sensitivity during food image exposure compared to both normal-weight and overweight HC
	Brooks et al. (2011) #	Imagining eating food shown in images or using non-food items	AN-R, AN-BP, BN, HC	50 females AN-R: 11, AN-BP: 7, BN: 8, HC: 24 mean age BN: 25 ± 7.1, mean age AN: 26 ± 6.8 mean age HC: 26 ± 9.5	BN: outpatient center, average duration of illness: 12 years, AN: Inpatient Unit, average duration of illness AN-R: 9 years, AN-BP: 9 years Antidepressant medication included	compared to HC: BN: ↓ bilateral superior temporal gyrus/insula + visual cortex;
	Olsavsky et al. (2019)#	Associative taste reward learning paradigm	AN-ill, AN-rec, BN, HC	111 females AN-ill: 28, AN-rec: 20, BN: 20, HC: 43 mean age AN-ill: 22.9 ± 5.0, mean age AN-rec: 30.0 ± 8.0, mean age BN: 25.3 ± 4.6, mean age HC: 26.4 ± 5.4	AN + BN: admitted to specialized treatment program, studied during first 1–2 weeks of treatment Antidepressants (14 AN-ill, 4 AN-rec, 13 BN), antipsychotics (3 AN-ill, 3 BN)	No difference to HC
	Jiang et al. (2019) #	Liking and wanting tasks using food odors	AN-R, BN, HC	39 females ANR: 14; BN: 13; HC: 12 mean age AN-R: 24.9 ± 4.7 mean age BN: 22.5 ± 2.9 mean age HC: 24.1 ± 3.1	AN-R: hospitalized BN: purging and binge-eating behaviors but ambulatory.	Wanting (motivational valuation) BN vs. HC ↓ Caudate, ventral pallidum, anterior insula Liking (hedonic evaluation) BN vs. HC ↔largely preserved Hunger modulation HC: ↑ activity in reward regions BN: ↓ blunted hunger modulation
Reward processing and subjective preferences	Donnelly et al. (2022)	Low vs. High Energy Food images (emotional categories: disgust, fear, happy) vs. non-food images	BN + BED vs. HC	38 females BN: 14; BED: 5; HC: 19 mean age BN&BED: 26.6 ± 13.0 mean age HC: 21.7 ± 1.2	BN + BED defined as one group: Hospitalized, psychological treatment HC: medically stable with no history of an ED and no current mental health diagnosis.	BN + BED: ↓ limbic, frontal, temporal, occipital activation for high-energy foods compared to HC temporal ↓ / occipital ↑ variable activation depending on emotion for low-energy foods - Binge frequency correlated with neural responses in temporal and occipital regions
	Chao et al. (2024)	Script-driven imagery task	BED, HC	40 females with BED (20 therapy group, 20 waiting list) mean age: 29.8 ± 7.1	BED: met DSM-5 criteria via structured interview. Mean BMI: 35.65 ± 7.14 Therapy group: ambulatory	No significant differences in BOLD responses between binge-eating scripts and neutral scripts in nucleus accumbens, dlPFC, OFC, ACC, and insula; neither within-group changes nor between-group changes from baseline to follow-up in Nucleus accumbens, dlPFC, OFC, ACC, or insula.
Disrupted reward learning and food choice	Simon et al. (2016)	Food incentive delay task	BN, BED, HC	111 females, BN: 56, BED: 27, HC: 55 mean age BN: 27.45 ± 10.55, mean age BED: 38.26 ± 13.75, mean age HC: 38 ± 10.85/25.74 ± 5.25	antidepressants (7 BN, 5 BED)	BN + BED vs. HC: ↑ Medial OFC during food reward receipt; ↑ in PCC, anterior medial PFC + angular gyrus BN + BED: ↑ medial OFC activation linked to ↑ food craving

Included studies compare findings from functional magnetic resonance imaging in patients with bulimia nervosa, binge-eating disorder with healthy controls. The table includes details on sample composition and illness characteristics. In the “Neuroimaging results” column, arrows are used to summarize group differences in brain activation or behavioral responses: an upward arrow (↑) indicates increased activation or preference in the patient group compared to controls, a downward arrow (↓) indicates decreased activation or preference, and a bidirectional arrow (↔) denotes no significant difference between groups. # A subgroup of patients with anorexia nervosa (AN) was included; the respective findings are summarized in Table 1. ACC, anterior cingulate cortex; dlPFC, dorsolateral prefrontal cortex; dmPFC, dorsomedial prefrontal cortex; ED, eating disorder; HC, healthy control; BN, bulimia nervosa; BED, binge eating disorder; PCC, posterior cingulate cortex; PFC, prefrontal cortex; OFC, orbitofrontal cortex; SMA, supplemental motor area.

Schienle et al. (2009) reported increased activation in the ACC and insula in participants with BN, compared to those with BED, as well as normal-weight and overweight controls, when viewing high-calorie food images. Notably, these responses were accompanied by heightened arousal and increased activation in the ACC and insula in BN, despite the images being rated as highly pleasant. This dissociation suggests a conflict between hedonic valuation and emotional reactivity, indicating a dysregulation in subjective preference processing in BN. Additionally, participants with BED showed enhanced medial OFC activity and stronger reward sensitivity during food image exposure compared to both normal-weight and overweight controls (Schienle et al., 2009). This pattern may reflect amplified subjective valuation of palatable food, aligning with behavioral tendencies toward binge episodes.

Brooks et al. (2011), who were also discussed in the AN section, examined neural responses to food images in women with BN using fMRI. In BN, greater activation was observed in the right dlPFC, cerebellum, and right precuneus compared with healthy controls, alongside engagement of reward-related and somatosensory regions. Deactivation was seen in the bilateral superior temporal gyrus/insula and visual cortex. These findings suggest that, in BN, heightened sensory and reward responsiveness may interact with cognitive control networks, potentially contributing to difficulties regulating intake and promoting binge-eating behaviors.

Overall, these findings suggest that BN and BED are characterized not only by heightened reward sensitivity but also by context-dependent and sometimes conflicting interactions between hedonic valuation and cognitive-emotional processing.

Olsavsky et al. (2019), also introduced in the AN section, employed an associative taste reward learning task to measure expected value signals during food reward prediction and included BN participants for comparison. BN participants did not show the heightened expected value signals in the ACC observed in acutely ill AN, suggesting that food-related reward processing in BN may be more strongly influenced by sensory and hedonic salience rather than predictive coding mechanisms.

Jiang et al. (2019) also identified distinct alterations in reward-related processing in BN, particularly during the motivational evaluation of food cues. Behaviorally, individuals with BN reported reduced desire to eat high energy-dense food in response to food odors, suggesting altered subjective wanting specifically for highly salient, calorie-dense stimuli. On the neural level, BN patients exhibited decreased activation of the caudate nucleus in response to food odors during hunger in the wanting condition compared to healthy controls. As the caudate is critically involved in goal-directed behavior and reward-based action selection, this attenuation may reflect impaired integration of motivational drive with behavioral output in food-related contexts. In addition, BN patients showed reduced activation in the anterior ventral pallidum and insula when exposed to high energy-dense food odors during hunger, indicating altered processing within striatal and interoceptive circuits central to reward valuation (Jiang et al., 2019).

Donnelly et al. (2022) examined neural responses in women with BN and BED to images of low- and high-energy foods categorized by emotional valence (disgust, fear, happy). Compared with healthy controls, participants with BN/BED showed differential activation to low-energy food images, with binge frequency correlating negatively with temporal lobe activity and positively with occipital lobe activity. For high-energy foods, neural responses were generally reduced across limbic, frontal, temporal, and occipital regions, whereas controls exhibited stronger activity (Donnelly et al., 2022). These findings suggest a potential neural “disengagement” from food stimuli, particularly for high-energy foods, which may reflect either anticipatory regulation of reward or altered engagement of self-control mechanisms. This study complements other work on passive reward processing in BN and BED, indicating that atypical neural activation patterns are sensitive to both the caloric content and emotional salience of food stimuli.

Chao et al. (2024) examined whether cognitive behavioral therapy (CBT) affects neural reward processing in BED. Participants underwent fMRI scanning while listening to personalized auditory scripts describing binge-eating or neutral-relaxing scenarios, a paradigm designed to probe cue-induced reward sensitivity. CBT led to clear reductions in binge-eating episodes and improvements in self-reported reward-driven eating, disinhibition, and hunger, indicating changes in subjective valuation and motivational drive (Chao et al., 2024). Interestingly, these behavioral improvements were not accompanied by significant changes in BOLD activation within typical reward-related brain regions. This suggests that, in BED, observed changes in eating behavior and subjective reward measures may not be immediately reflected in neural activation patterns. However, the authors note that further research is needed to clarify the mechanisms linking CBT-induced behavioral improvements to reward system functioning.

Taken together, these studies indicate that reward processing in BN and BED is highly state- and stimulus-dependent, with evidence for both enhanced hedonic responsivity and context-specific disengagement of reward-related neural systems.

In addition to subjective food valuation, a distinct aspect of reward processing in EDs has been examined in patient studies using choice-based and reward prediction paradigms, which probe how neural and behavioral responses to food stimuli guide decision-making. For example, Simon et al. (2016) investigated neural signatures of food reward processing in BN and BED. Using event-related fMRI, they found that controls exhibited higher activation in the posterior parietal cortex during food reward anticipation compared to participants with BN and BED in whole-brain analyses. During the receipt of high compared to no food rewards, BN and BED participants showed heightened activation in the medial OFC than controls, according to region-of-interest analyses. Additionally, whole-brain results indicated greater activation in the PCC, anterior medial PFC, and angular gyrus among those with BN and BED compared to controls. These effects were specific to food rewards, as no significant group differences were observed for monetary rewards. Increased medial OFC activation during food receipt was associated with higher levels of food craving and external eating behaviors in participants with ED, suggesting that BN and BED are rather characterized by an enhanced hedonic response to food rewards, in contrast to AN, where reward processing may be impaired (Simon et al., 2016).

Taken together, individuals with BN and BED exhibit altered reward processing and subjective responses to food cues. Neural responses are particularly sensitive to the hedonic and motivational salience of foods, with heightened activation in reward-related and sensory regions during the receipt of palatable stimuli, while some highly salient, high-energy foods may elicit reduced or disengaged responses. This pattern reflects a dissociation between hedonic valuation, motivational drive, and top-down regulatory processes, and highlights how altered subjective preferences contribute to binge-eating behavior. Cognitive control and associative learning networks are variably engaged, suggesting that immediate sensory and motivational responses may dominate over learned reward predictions. Behavioral interventions can reduce binge episodes and modify subjective reward-driven eating, although neural changes may lag behind, indicating that altered subjective valuation and motivational processing may precede measurable changes in brain activity. Collectively, these findings underscore that BN and BED are characterized by enhanced hedonic responsiveness, dysregulated motivational signaling, and complex interactions between reward sensitivity, cognitive control, and self-regulation.

## 4 Discussion

Altered food valuation in EDs is not limited to simple hedonic pleasure but involves complex subjective preferences shaped by experience, emotion, and cognitive control. These distortions may reinforce avoidance, restrictive patterns, or binge-eating behaviors. By framing EDs in terms of disrupted subjective valuation, we can link neural reward mechanisms to observable eating behavior, highlighting both the psychological and neurobiological dimensions of the disorder. These biases may generalize to other domains, suggesting broader dysfunctions in valuation mechanisms across contexts.

Building on these insights, altered subjective preferences have direct clinical relevance. They may contribute to disordered eating behaviors, such as avoidance, binge eating, or restrictive patterns, by reinforcing maladaptive reward responses. As the studies discussed in the present review illustrate, reward processing in EDs is not merely about how individuals respond to food; it also reflects broader patterns of neural dysfunctions that affect behavior across various domains.

Situating subjective food valuation within established theoretical frameworks, such as neurobiological reward deficit models (Frank, 2013), reward prediction error accounts (Frank et al., 2021; Schaefer and Steinglass, 2021), and wanting-liking dissociation models (Morales and Berridge, 2020), highlights how preference-based paradigms may help refine these accounts. Whereas reward deficit models emphasize altered striatal responsivity, and prediction error models focus on aberrant reinforcement learning signals, neither framework explicitly specifies how these neural alterations translate into consciously experienced subjective value. Direct assessment of food cue valuation may therefore represent an intermediate phenotype linking neurobiological reward mechanisms to observable eating behavior. Therapies that address maladaptive subjective preferences and food-related decision-making could offer additional benefit. While food exposure is already a core component of CBT for EDs, the approaches suggested here diverge by explicitly targeting underlying neural and cognitive mechanisms implicated in reward processing. By incorporating knowledge from studies of wanting-liking dissociation, reward prediction errors, and striatal responsivity (Morales and Berridge, 2020; Frank et al., 2021; Schaefer and Steinglass, 2021), these interventions aim to calibrate subjective valuation and decision-making processes that maintain maladaptive eating behaviors. For example, exposure tasks could be adapted to systematically shift anticipatory “wanting” or to correct maladaptive prediction error learning, rather than solely focusing on behavioral habituation or anxiety reduction. Similarly, cognitive training and neurofeedback approaches could be designed to modify how the brain encodes and updates food-related value representations, offering a more mechanistically grounded complement to standard CBT.

Potential interventions could include, but are not limited to:

- Exposure to preferred and non-preferred foods while monitoring subjective responses to assess and shift maladaptive food-related preferences, targeting anticipatory “wanting” versus consummatory “liking.”
- Cognitive training programs designed to target reward valuation and decision-making biases, including maladaptive prediction error learning, with a focus on how individuals with EDs assign value to food stimuli and other relevant choices.
- Neurofeedback, neuromodulation, or event-related potential-based interventions aimed at normalizing reward-related brain responses, such as targeting altered P300 amplitudes (an EEG marker) in response to food stimuli, which could help recalibrate the reward systems and improve food-related decision-making.

Incorporating these approaches into treatment research could not only target the physiological aspects of disordered eating but also address the deeper, cognitive-emotional distortions in food valuation that underlie maladaptive behaviors. Future research should investigate the efficacy of these interventions, ideally using neuroimaging techniques to assess changes in brain activation and connectivity related to reward processing.

### 4.1 Limitations of current research

Despite these insights, several important limitations of the current literature should be noted. Most studies focus exclusively on adult female participants, leaving sex differences and developmental trajectories in subjective valuation underexplored. Methodologically, fMRI dominates the current literature, providing high spatial but limited temporal resolution; EEG and multimodal approaches remain scarce in ED populations. Most studies are cross-sectional or short-term pre/post designs, limiting understanding of how subjective valuation and reward processing change over time or with treatment. Behavioral markers, such as food choice patterns, may provide useful prognostic information, even when neural or subjective changes are slow to emerge (Steinglass et al., 2024). Future research should expand participant diversity, incorporate multimodal imaging (EEG-fMRI), and use longitudinal designs to track the dynamics of reward processing and subjective food valuation across illness stages and recovery trajectories. Preference-based, devaluation, and decision-modulation paradigms could be applied to investigate anticipatory “wanting,” consummatory “liking,” prediction error learning, and learned value representations, linking neural mechanisms to clinically relevant behaviors. Integrating these approaches may not only clarify disorder-specific versus dimensional alterations in subjective valuation but also inform the development of mechanistically grounded interventions that target the cognitive-emotional processes maintaining maladaptive eating behaviors.

Limitations of the present review include its narrative, non-systematic approach to literature selection, which may have introduced selection bias and limited reproducibility compared to systematic review methodologies. However, this approach was chosen to allow for a conceptually driven synthesis across heterogeneous theoretical, behavioral, and neuroimaging findings.

## 5 Conclusion

Aberrant reward processing appears to be a central feature of EDs and may act as a transdiagnostic factor across subtypes. This altered processing is closely linked to changes in how individuals subjectively value food, contributing to the complex psychopathology of EDs. Neuroimaging research consistently demonstrates that foods typically perceived as pleasant can paradoxically trigger anxiety or avoidance in EDs, highlighting disrupted valuation mechanisms. Emerging evidence from healthy populations further shows that subjective food preferences are flexible and shape neural responses depending on factors such as hunger, personal preference, and choice. Recognizing these altered food preferences in EDs and their underlying neural mechanisms may inform future research and guide the development of more personalized and effective treatments.
